# The Role of a Dipeptide Transporter in the Virulence of Human Pathogen, *Helicobacter pylori*

**DOI:** 10.3389/fmicb.2021.633166

**Published:** 2021-02-25

**Authors:** Xiaohong Xu, Junwei Chen, Xiaoxing Huang, Shunhang Feng, Xiaoyan Zhang, Feifei She, Yancheng Wen

**Affiliations:** ^1^Key Laboratory of Gastrointestinal Cancer (Fujian Medical University), Ministry of Education, Fuzhou, China; ^2^Fujian Key Laboratory of Tumor Microbiology, Department of Medical Microbiology, Fujian Medical University, Fuzhou, China; ^3^Fujian Medical University Union Hospital, Fuzhou, China

**Keywords:** *Helicobacter pylori*, dipeptide transporter, NF-κB, T4SS, outer membrane proteins

## Abstract

*Helicobacter pylori* harbors a dipeptide (Dpp) transporter consisting of a substrate-binding protein (DppA), two permeases (DppB and C), and two ATPases (DppD and F). The Dpp transporter is responsible for the transportation of dipeptides and short peptides. We found that its expression is important for the growth of *H*. *pylori*. To understand the role of the Dpp transporter in the pathogenesis of *H*. *pylori*, the expression of virulence factors and *H*. *pylori*-induced IL-8 production were investigated in *H. pylori* wild-type and isogenic *H. pylori* Dpp transporter mutants. We found that expression of CagA was downregulated, while expression of type 4 secretion system (T4SS) components was upregulated in Dpp transporter mutants. The DppA mutant strain expressed higher levels of outer membrane proteins (OMPs), including BabA, HopZ, OipA, and SabA, and showed a higher adhesion level to gastric epithelial AGS cells compared with the *H*. *pylori* 26695 wild-type strain. After infection of AGS cells, *H*. *pylori* Δ*dppA* induced a higher level of NF-κB activation and IL-8 production compared with wild-type. These results suggested that in addition to supporting the growth of *H*. *pylori*, the Dpp transporter causes bacteria to alter the expression of virulence factors and reduces *H*. *pylori*-induced NF-κB activation and IL-8 production in gastric epithelial cells.

## Introduction

*Helicobacter pylori* is a microaerophilic, Gram-negative bacterium that is closely related to chronic gastritis, peptic ulcers, and gastric cancer (Kusters et al., 2006; Chmiela and Kupcinskas, 2019). To colonize the human stomach, *H. pylori* has to pass through the mucous layer to the surface of gastric mucosal epithelial cells via the movement of its flagella, and then colonizes the epithelial cells with the aid of adhesins (Sgouras et al., 2015). The pathogenesis of *H. pylori* is driven by several virulence factors that facilitate bacterial colonization, induce inflammation, and damage host cells. Among the virulence factors confirmed to function in *H*. *pylori* infection, Type 4 secretion system (T4SS) and its effector protein CagA encoded by *cag* pathogenicity island (*cag*PAI) are one of the most extensively studied *H. pylori* virulence factors (Guillemin et al., 2002; Sanchezzauco et al., 2013). *cag*PAI is about 40 kb in size and comprises 26 genes that encode the components of the T4SS. Relying on the T4SS, which binds to the α5β1 integrin expressed on the surface of gastric epithelial cells (Kwok et al., 2007), *H. pylori* delivers CagA, ADP-heptose (Pfannkuch et al., 2019), and peptidoglycan into host cells (Viala et al., 2004). *H. pylori* infection activates nuclear factor-kappa B (NF-κB) in gastric epithelial cells, inducing the release of proinflammatory factors such as interleukin 8 (IL-8) (Maeda et al., 2000; Backert and Naumann, 2010).

Successful colonization requires adaptation of the bacterium to the gastric environment. Environmental factors such as pH, reactive oxygen species, temperature, or nutrients can affect the expression of *H. pylori* virulence factors (Merrell et al., 2003a; Pflock et al., 2006; Augusto et al., 2007; Noto et al., 2015). Acidic pH highly stimulates the expression of antioxidant proteins, flagellar structural proteins, and T4SS component proteins in *H*. *pylori* (Marcus et al., 2018). Upregulation of *vacA* and downregulation of genes related to motility were observed under iron-restricted conditions (Merrell et al., 2003b). Iron deficiency enhances *H. pylori* virulence; thus, *H. pylori* isolated from iron-depleted gerbils expressed significantly higher levels of CagA, which induced more robust proinflammatory responses (Noto et al., 2012).

Considering that nutrients are important for the growth of bacteria, genes involved in the metabolism serve as targets for antimicrobial therapies. The peptide transporter systems have been extensively investigated in bacteria such as *Escherichia coli* and *Lactococcus lactis* (Sanz et al., 2001; Harder et al., 2008). Peptide transporters play an important role in nutritional supply by providing carbon sources or nitrogen sources for bacterial growth (Gilvarg, 1972). Three types of peptide transporters in bacteria have been found to date: oligopeptide (Opp) transporters, dipeptide (Dpp) transporters, and dip/tripeptide (Dtp) transporters (Garai et al., 2017). The Opp and Dpp transporters belong to the ATP-binding cassette (ABC) superfamily, while the Dtp transporter belongs to the proton-dependent oligopeptide transporter (POT) (Paulsen and Skurray, 1994). The Dpp transporters are responsible for transporting mainly dipeptides but also tripeptides into cells (Payne and Smith, 1994), while the Opp transporters are responsible for the import of oligopeptides. The Dpp transporter in *H*. *pylori* is composed of five proteins encoded by *dppA, B, C, D*, and *F* (Davis and Mobley, 2005; Weinberg and Maier, 2007). DppA is a periplasmic peptide-binding protein, DppB and DppC are integral membrane proteins that form permeases for substrates, while DppD and DppF are cytoplasmic proteins responsible for ATP hydrolysis.

Apart from being involved in the transport of nutrients, peptide transporters play a role in the virulence of various bacterial pathogens (Samen et al., 2004; Moraes et al., 2014). In *E. coli*, the Dpp transporter acts as a primary chemoreceptor, and its interaction with the membrane components for dipeptide chemotaxis initiates flagellar motion (Manson et al., 1986). In *Borrelia burgdorferi*, an *opp* mutant strain promotes the expression of the virulence factor OspC by regulating the Rrp2-RpoN-RpoS pathway (Zhou et al., 2018). In group A *Streptococci*, Dpp mutation results in a decreased expression of SpeB, a major cysteine protease (Podbielski and Leonard, 1998). In *Pseudoalteromonas*, DppA plays an important role in cold adaptation (Zhang et al., 2010). However, hitherto the role of Dpp transporters in the growth and pathogenesis of *H. pylori* remains unknown. A study has shown that expression of DppA in *H*. *pylori* was stimulated by gastric epithelial cells, suggesting that DppA might play an important role in the pathogenesis of *H*. *pylori* (Sharma et al., 2010).

In this work, we constructed Dpp transporter mutants in *H*. *pylori* and evaluated the effects of the Dpp system on growth, expression of virulence factors, and inflammatory responses of AGS cells stimulated by *H*. *pylori*.

## Materials and Methods

### Bacterial Strains and Cultivation Conditions

*Helicobacter pylori* 26695, NCTC11637 and Dpp transporter mutant strains were cultured in a microaerobic environment (5% O_2_, 10% CO_2_, and 85% N_2_) at 37°C on Columbia agar plates (Oxoid, Cambridge, United Kingdom) containing 7% sheep blood. For liquid cultivation of *H*. *pylori*, Brucella broth supplied with 10% fetal bovine serum (FBS) was used, and the strains were incubated in a shaker at 120 rpm and 37°C. A total of 5 μg/ml kanamycin (MP Biomedicals, CA, United States) was supplied when necessary.

### Construction of Isogenic Δ*dppA*, Δ*dppB*, Δ*dppC*, Δ*dppD*, Δ*dppF* Mutants of *H. pylori* 26695 and Isogenic Δ*dppA* Mutant of NCTC11637

To construct a *dppA* knockout mutant of *H. pylori* 26695 (Δ*dppA*), a DNA fragment containing an upstream sequence of *dppA* was amplified with the primers DppA-up-F and DppA-up-R, a DNA fragment containing a downstream sequence of *dppA* was amplified with the primers DppA-down-F and DppA-down-R, and a DNA fragment containing AphA, which confers kanamycin resistance, was amplified with primers DppA-Kana-F and DppA-Kana-R. The *dppA* upstream sequence, *dppA* downstream sequence, and kanamycin resistance DNA fragments were ligated into a pBluescript II SK (–) vector (Novagen, Madison, WI, United States) using the ClonExpress MultiS One Step Cloning Kit (Vazyme, Nanjing, China), resulting in pBluscript-DppAKO, which was further transformed into *E. coli* DH5α. The plasmid sequence was then confirmed using colony PCR and Sanger sequencing. The pBluescript-DppAKO was then purified and subsequently transformed to *H. pylori* 26,695 by electroporation, and bacteria were then cultivated on agar plates containing kanamycin. *dppA* knockout mutants were further confirmed by colony PCR and Sanger sequencing. The construction of isogenic *H. pylori* 26695 mutants of Δ*dppB*, Δ*dppC*, Δ*dppD*, Δ*dppF*, and NCTC11637Δ*dppA* was conducted in a similar manner, and the primers used are listed in Table 1.

**TABLE 1:** Primers used in this study.

Primers	Sequence (5′–3′)
**For construction of isogenic mutants in *H. pylori* 26,695**	
DppA-up-F	AGGGCGAATTGGGTACCGAATTAGTGGAAGTGTTAG CCGTAT
DppA-up-R	AACCGCCCAGTCTCGAGGATGGATTAAGAGT AGCGTTTGG
DppA-down-F	CATTTTTAATTTCTCGAGTACCCACTCAAAACC ATTCCTT
DppA-down-R	GTGGCGGCCGCTCTAGATAAATCAGCATGAGCC CTAGCC
DppA-Kana-F	CTCGAG ACTGGGCGGTTTTATGGACAGC
DppA-Kana-R	CTCGAG AAATTAAAAATGAAGTTTTAGC
DppB-up-F	TATAGGGCGAATTGGGTACCtGCGATCAATGCAG ATGATTACATC
DppB-up-R	AAAACCGCCCAGTAAACAGCGTGGGGATCGC
DppB-down-F	ATTTTGATGTATATTGGGGCTAATCTCTTAG
DppB-down-F	AGGAATTCGATATCAAGCTTTGGGAGGTTGGGCCCCAA
DppB-Kana-F	GCTGTTTACTGGGCGGTTTTATGGACA
DppB-Kana-F	GCCCCAATATACATCAAAATTAAAAATGAAGTTTTAGCAC GTG
DppC-up-F	TATAGGGCGAATTGGGTACCTTTGGCAACGCTTCCCGG
DppC-up-R	GTCCATAAAACCGCCCAGTTTTTGAATTGTTGGATAAAC TCTCTAAA
DppC-down-F	TTGGATGCTTGTTTTCCCTGG
DppC-down-R	AGGAATTCGATATCAAGCTTACCACGCCTAAA TCATG GGTG
DppC-Kana-F	AACTGGGCGGTTTTATGGACA
DppC-Kana-R	CAGGGAAAACAAGCATCCAAAAATTAAAAATGA AGTTTTAGCACGTG
DppD-up-F	TATAGGGCGAATTGGGTACCCGCCTTTTGAAGCCT ATATGGG
DppD-up-R	GCCCAGTCTTATCGGTGAAAAAATAAGTTTTTAAAT
DppD-down-F	TAATTTGGATGAAAATGTGGATTATTTGAGTT
DppD-down-R	AGGAATTCGATATCAAGCTTGCGCTTATCACGC TATCCACA
DppD-Kana-F	TTTCACCGATAAGACTGGGCGGTTTTATGGACA
DppD-Kana-R	CCACATTTTCATCCAAATTAAAAATGAAGTTTT AGCACGTG
DppF-up-F	TATAGGGCGAATTGGGTACCAGGGTTGATTGAAA AACCGGG
DppF-up-R	CGCCCAGTTTAGGCTTGAATAACCCCCTGTC
DppF-down-F	TCCAAAGCACCCTTATACGCA
DppF-down-R	AGGAATTCGATATCAAGCTTTAAGCCCTTTCC CTCGCTAGC
DppF-Kana-F	ATTCAAGCCTAAACTGGGCGGTTTTATGGACA
DppF-Kana-R	GCGTATAAGGGTGCTTTGGAAAATTAAAAATGA AGTTTTAGCACGTG
**For construction of isogenic mutant in NCTC11637**	
DppA-up-F	TATAGGGCGAATTGGGTACCACTATAATAAGCGTTTA TTTTAAAAAGAGCG
DppA-up-R	AAAACCGCCCAGTCATAAGCCAGTCTCCACAACAAAT
DppA-down-F	TAGCCTATCCTTATTCGGTGGTG
DppA-down-R	AGGAATTCGATATCAAGCTTAGTGCTTGATCGTATC CATAAACG
DppA-Kana-F	GCTTATGACTGGGCGGTTTTATGGACA
DppA-Kana-R	CACCGAATAAGGATAGGCTAAAATTAAAAATGAAG TTTTAGCACGTG
**For qPCR**	
Cagδ-F	GTGCTATGGGGATTGTTGGGATA
Cagδ-R	TTGCTTGAGATTTTTGAGTTTCG
CagV-F	GGCTTTTTATCTCTCTATGGCACTC
CagV-R	CAATTTTAAATTCTCCTGTGTATCG
CagU-F	AAAAGCTACCGCAAGAAAAAAGG
CagU-R	AAACAAAACAAATATCCCACCCA
CagS-F	CAAGGGAGCGTTAGATAAGGTTCT
CagS-R	AATTAGGATTCTCTGCAATGGCAT
CagQ-F	CCGAACAAGCAAGAACTTACACAAC
CagQ-R	TCATTAACATCAGGAAGAACAAAAA
CagP-F	ACAATTCAAACATTCTTTCAACAA
CagP-R	GATAAACTAAAATCACCCCTGCCC
CagM-F	AAACAAATACAAAAAAGAAAAAGAGG
CagM-R	AAACATAGGCATAAGGGTTAGGAAGA
CagC-F	GGGTCAAAGGCATAGCGGATAT
CagC-R	GAAGCCAAACTTAGTGCTCAAA
CagA-F	GCCACTACTACCACCGACATACAAGG
CagA-R	GTCAGCGACTCCCTCAACATCTAACA
CagL-F	TGCTGAGCAACAATGCGGAATATCC
CagL-R	GCGTCTGTGAAGCAGTGATTAAGGAA
16s rRNA-F	GGCGACCTGCTGGAACATTACTGAC
16s rRNA -R	CCAGGCGGGATGCTTAATGCGTTAG
AlpA-F	CGGTGCGACTGGTTCAGATGGT
AlpA-R	AGCGGCTACGGCAGAGTTGAAA
AlpB-F	GGCTTACGCTACTACGGCTTCTTCA
AlpB-R	CCCGCATTAAGACTTCGGCTACCAA
HopZ-F	GCAAACACGCAAGGGCTGATTGG
HopZ-R	CTCTTACCAGGACCGCATTGGACAT
BabA-F	GCACTGGTGGCACACAAGGTTCA
BabA-R	CGGCTTGCTGTATCTGCTGCTCTT
SabA-F	GCACCACCCAATCGCCCATCTTTA
SabA-R	ACACTAGCGGGTTGCCCACTATCA
HopQ-F	GCGTTGAGATCGGTGTTAGGGCTAT
HopQ-R	TGCCATTGCCATTCTCATCGGTGTA
HpaA-F	GAGAGCGATGCGCTTAGCGAAGA
HpaA-R	CGCCGCAATTCCACTCTTTCAATCA
OipA-F	GCCGATTCGCAGGAAATGGTGGA
OipA-R	AACCGCTACCAGGAACAGAACCAAC
SabB-F	CCACTGGTCCTGTAACCGACTATGC
SabB-R	GAGATCCTGTGGCTTGAGCTTGCA
IL-8-F	GAAGGTGCAGTTTTGCCAAG
IL-8-R	TTTCTGTGTTGGCGCAGTG
GAPDH-F	AAATTCCATGGCACCGTCAAG
GAPDH-R	GGACTCCACGACGTACTCAG

*Underlined part indicates overlapping DNA sequences.*

### Cell Lines, Cultivation, and Co-culture of AGS Cells and *H. pylori* Strains

The human gastric epithelial AGS cell line (derived from a human gastric adenocarcinoma) was cultured in a DMEM/F12 medium (HyClone Laboratories Inc., Logan, UT, United States), with supplementation of 10% FBS (PANS, Aidenbach, Bayern, Germany) at 37°C in a 5% CO_2_ humidified atmosphere. For *H*. *pylori* infection assays, AGS cells were grown in 6-well plates (NUNC, Thermo, DE, United States) until the confluence reached 75% in DMEM/F12 medium containing 10% FBS. Before infection, the supernatant was removed, and cells were washed twice with phosphate-buffered saline (PBS), followed by culture in FBS-free DMEM/F12 for 4 h. *H*. *pylori* strains were first cultivated on agar plates; then, the bacteria were collected and resuspended in Brucella broth at an initial OD_600_ of 0.1, followed by culture for 24 h. Bacterial cells were then pelleted and washed twice with DMEM/F12 medium, resuspended in DMEM/F12 medium, and added to the AGS cell culture at a multiplicity of infection (MOI) of 100.

### Determination of Bacterial Growth Rates

To monitor the growth of *H*. *pylori* strains, bacteria were first cultivated on Columbia agar plates for 3 days, followed by collection of bacterial cells and resuspension in Brucella broth at an initial OD_600_ = 0.1. Next, the bacteria were cultured at 37°C with agitation. The OD_600_ values of the bacterial culture were recorded every 8 h. Each experiment was repeated at least three times.

### RNA Sequencing and Data Analysis

To prepare total RNA for transcriptomic study, *H. pylori* 26,695 and Δ*dppA* cells were cultivated in Brucella broth containing 10% FBS for 20 h until reaching the exponential phase in a shaker at 120 rpm in a microaerobic environment (5% O_2_, 10% CO_2_, and 85% N_2_) at 37°C. Total RNA was isolated using the RNeasy Mini Kit (QIAGEN, Valencia, CA, United States). RNA degradation and contamination were monitored on 1% agarose gels. RNA sequencing was carried out at Novogene (Beijing, China), and RNA purity was confirmed using a NanoPhotometer spectrophotometer (IMPLEN, CA, United States). RNA concentration was measured using the Qubit RNA Assay Kit with a Qubit 2.0 Flurometer (Life Technologies, CA, United States). RNA integrity was assessed using the RNA Nano 6000 Assay Kit for the Agilent Bioanalyzer 2100 system (Agilent Technologies, CA, United States). Ribosomal RNA (rRNA) was then depleted using the Ribo-zero kit (Ambion, Thermo, DE, United States) in accordance with the manufacturer’s instructions. Sequencing libraries were generated using the NEBNext Ultra Directional RNA Library Prep Kit for Illumina (NEB, Ipswich, MA, United States) following the manufacturer’s recommendations, and index codes were added to attribute sequences to each sample. The clustering of the index-coded samples was performed on a cBot Cluster Generation System using the TruSeq PE Cluster Kit v3-cBot-HS (Illumina, San Diego, CA, United States). After cluster generation, the library preparations were sequenced on an Illumina Hiseq platform, and paired-end reads were generated. The resulting *P*-values were adjusted using the Benjamini and Hochberg’s approach for controlling the false discovery rate. Genes with an adjusted *P*-value < 0.05 found by DESeq were designated as differentially expressed. The data were deposited in the NCBI Gene Expression Omnibus database (GEO^1^) under accession number GSE164216.

### RNA Isolation and Quantitative RT-PCR

To prepare bacterial RNA samples, bacteria were grown in Brucella broth containing 10% FBS for 20 h, which was followed by extraction of total RNA using a RNeasy Mini Kit (QIAGEN, Valencia, CA, United States) in line with the manufacturer’s instructions. In order to extract RNA from AGS cells or AGS cells infected with *H*. *pylori*, the cells were collected after co-culture with bacteria at 37°C in a 5% CO_2_ humidified atmosphere after infection with *H. pylori* using TRIzol reagent (Life Technologies, Carlsbad, CA, United States) according to the manufacturer’s instructions. RNA concentration and purity were then determined by spectrophotometry (NanoDrop One, Thermo, DE, United States). For quantitative RT-PCR (qPCR) analysis, cDNA was prepared through reverse transcription using 1 μg of total RNA and the HiScript II Q RT SuperMix for qPCR (+gDNA wiper) kit (Vazyme, Nanjing, China). qPCR assays were carried out using the SYBR qPCR Master Mix kit (Vazyme, Nanjing, China). Specific primers for each gene indicated were designed with Primer 5.0 and are listed in Table 1. Genes encoding 16S rRNA were used as endogenous controls, and relative RNA levels were calculated using the 2^–ΔΔ*Ct*^ method. Experiments were performed in triplicate for each condition.

### Bacterial Pulldown Assays

*Helicobacter pylori* was grown in Brucella broth containing 10% FBS for 20 h, after which the bacterial cells were washed twice and resuspended in PBS. Approximately 3 × 10^7^ cells were incubated with α5β1 integrin (250 μg/mL) (R&D Systems, Minneapolis, MN, United States) for 30 min at 37°C with rotation. In order to measure the amount of α5β1 integrin bound by *H*. *pylori*, the samples were centrifuged at 6,000 rpm for 10 min. Bacterial cells were collected, washed twice with PBS, and then resuspended in 1 × SDS loading buffer (60 mM Tris-HCl [pH 6.8], 2% SDS 10 ml, 10% glycerol, 100 mM DTT, 0.01% bromophenol blue). After denaturation by boiling for 10 min, the samples were resolved on a 10% SDS-polyacrylamide gel (SDS-PAGE). Western blot with an anti-β1 Rabbit antibody (1:1000; Cell Signaling Technology, Danvers, MA, United States) was performed as described previously.

### Bacteria Protein Extraction and Western Blotting

To determine the expression of CagA, *H*. *pylori* cells were harvested after 20 h culture in Brucella broth containing 10% FBS. The cells were then washed twice with PBS, and total lysates were obtained using RIPA lysis buffer (Beyotime, Shanghai, China). The concentration of proteins was determined using a BCA Protein Assay Kit (Beyotime, Shanghai, China). Protein samples were then subjected to electrophoresis using a 10% SDS-PAGE gel and subsequently transferred to PVDF membranes (Millipore, Darmstadt, Germany) for antibody blotting. Membranes were blocked with Tris buffered saline containing 0.1% Tween-20 (TBST) containing 5% BSA. The membranes were then probed with an anti-CagA (b-10) antibody (1:800; Santa Cruz Biotechnology, Dallas, TX, United States), followed by an m-IgGκBP-HRP secondary antibody (1:1500; Santa Cruz Biotechnology, Dallas, TX, United States).

### Adhesion Tests

AGS cells were seeded in 6-well plates at a density of 3.5 × 10^5^ cells/well with 2 ml of DMEM/F12 to form a confluent monolayer, and then infected with *H. pylori* at an MOI of 100 as described above. After 4 h of infection, the AGS cells were washed three times with PBS to remove any unattached bacteria. To determine the number of adherent *H*. *pylori*, the AGS cells were lysed using 0.1% saponin for 20 min at room temperature. After a serial dilution, 50 μl of each diluted cell lysate containing bacteria was placed on a Columbia sheep blood plate. Subsequently, the bacteria were incubated under microaerobic condition (5% O_2_, 15% CO_2_, and 75% N_2_) for 4 days, and colonies were counted.

### Dual-Luciferase Reporter Assay

NF-κB activation was determined using luciferase reporter assays. AGS cells were seeded in 12-well plates at a density of 5 × 10^5^ cells/well in 1 ml DMEM/F12 with FBS and cultured overnight. Subsequently, 1 μg of pNL3.2.NF-κB-RE (Promega, Madison, WI, United States) and 0.1 μg of pRL-TK (Promega, Madison, WI, United States) were co-transfected into these AGS cells using Lipofectamine 3000 (Invitrogen, Carlsbad, CA, United States) following the manufacturer’s instruction. After 48 h of culture, the cells were infected with *H. pylori* strains at an MOI = 100. After infection for 4 h, the AGS cells were harvested, and luciferase activities were measured using the Dual-Luciferase Reporter Assay System (Promega, Madison, WI, United States) in accordance with the manufacturer’s instruction. Each result represents the mean of three independent experiments.

### IL-8 Secretion Assays

The AGS cells were seeded in 6-well culture plates at a density of 3.5 × 10^5^ per well in 2 ml DMEM/F12 medium with FBS to form a confluent monolayer. After 20 h of culture, the supernatant was replaced with fresh DMEM/F12 without serum after washing with 1 × PBS to starve cells for 4 h. Next, the cells were infected with *H. pylori* 26,695 at an MOI of 100 or infected with NCTC11637 at an MOI of 30. After 4 h of infection, the supernatant was harvested, and IL-8 concentration was measured using enzyme linked immunosorbent assay (ELISA) with a Human IL-8 ELISA kit (BD Biosciences, San Jose, CA, United States) in line with the manufacturer’s instructions.

### Statistical Analysis

All data were presented as the mean ± the standard error of mean. An unpaired *t*-test was used for comparisons between the two groups. Graph-Pad Prism 7.0 (La Jolla, CA, United States) was used to plot the data, and *P* < 0.05 was considered statistically significant.

## Results

### Dpp Transporters Are Important for the Growth of *H. pylori*

**FIGURE 1:**
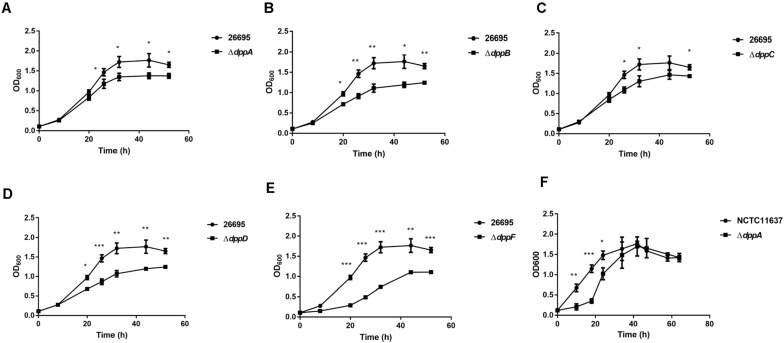
Growth of *H. pylori* wild-type strains and Dpp transporter mutant strains. Growth curves of *H*. *pylori* 26695 compared to its isogenic mutants Δ*dppA* **(A)**, Δ*dppB* **(B)**, Δ*dppC* **(C)**, Δ*dppD* **(D)**, and Δ*dppF* **(E)**. **(F)** Growth curve of *H. pylori* NCTC11637 compared with its isogenic mutant Δ*dppA*. Data shown represent average means from three independent experiments, and standard deviations are also indicated. ***P < 0.001, **P < 0.01, *P < 0.05.

Five genes were studied, *dppA, B, C, D*, and *F*, which encode a dipeptide (Dpp) transporter comprising two permeases, two ATPases, and a substrate-binding protein (Davis and Mobley, 2005). To study the role of Dpp transporters in the virulence of *H*. *pylori*, we first constructed isogenic mutants of *dppA*, *dppB*, *dppC*, *dppD*, and *dppF* in *H. pylori* 26,695, and then assessed the impact of the Dpp transporter on bacterial growth. Growth curves showed that *H. pylori* 26,695 Δ*dppA*, Δ*dppB*, Δ*dppC*, Δ*dppD*, and Δ*dppF* strains proliferated slower than a wild-type strain (Figures 1A–E). Specifically, *DppF* had the strongest effect on the growth of *H*. *pylori*, suggesting its important role in the bacterial growth. We also constructed a Δ*dppA* mutant in the *H*. *pylori* strain NCTC11637 and conducted the same experiment. The Δ*dppA* mutant grew slower compared with the NCTC11637 wild type (Figure 1F). The results indicated that the importance of the Dpp system for the growth of *H*. *pylori*.

### Transcriptomic Profiling of Gene Expression in *H*. *pylori* Wild-Type and Δ*dppA* Strains

**FIGURE 2:**
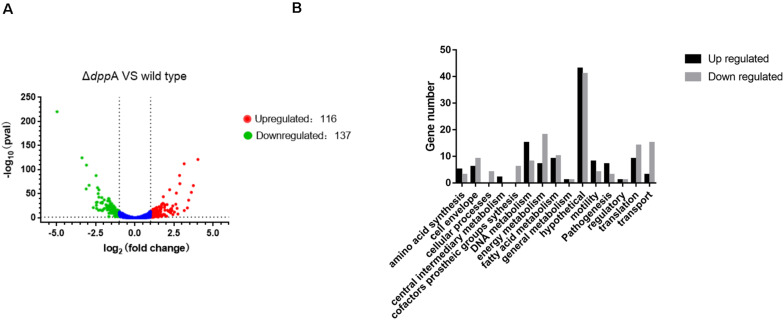
Differentially expressed genes between *H*. *pylori* 26695 and Δ*dppA* by RNA sequencing. **(A)** Volcano plot of gene expression in *H*. *pylori* 26695 and Δ*dppA*. The *Y*-axis represents -log_10_ (*P*-value), and *X*-axis represents log_2_ (fold change). Positive values represent genes upregulated in Δ*dppA*, while negative values represent genes downregulated in Δ*dppA*. The horizontal dashed line represents *P* = 0.05. Red dots represent those genes with expression in Δ*dppA* higher than wild type, with Log_2_ (fold change) > 1 and *P* < 0.05. Green dots represent genes with lower expression in Δ*dppA* compared with wild-type, with Log_2_ (fold change) < –1 and *P* < 0.05. **(B)** Functional annotation of genes differentially expressed in *H*. *pylori* 26,695 and Δ*dppA*. Black bars represent genes expressed higher in Δ*dppA* compared with wild type, while gray bars represent genes expressed lower in Δ*dppA* compared to wild type.

To further examine the roles of DppA, we performed RNA-seq analysis and investigated the genes expressed differentially between *H*. *pylori* 26695 and a Δ*dppA* strain. We found that 253 genes were differentially expressed with a | Log_2_ (fold change)| > 1, including 116 genes that were upregulated and 137 genes that were downregulated in Δ*dppA* (*P* < 0.05) (Figure 2A). These genes are listed in Table 2. We also performed functional classification of the genes upregulated and downregulated in Δ*dppA* (Figure 2B). We found that genes involved in energy metabolism, cellular processes, transportation, and translation were significantly downregulated in Δ*dppA*, which might contribute to the decreased growth rate of *H*. *pylori* in this genetic background. Genes involved in DNA metabolism, bacterial pathogenesis, and motility were upregulated in this strain, and they might contribute to the virulence of *H*. *pylori*.

**TABLE 2:** Differentially expressed genes identified by RNA-seq.

Gene expression	Gene no.	Gene name or function	log_2_ (fold change)
Upregulated	HP0059	Predicted gene	2.4264
	HP0114	Predicted gene	1.3287
	HP0115	flaB	1.7312
	HP0116	topA	1.378
	HP0117	Predicted gene	1.3026
	HP0119	Predicted gene	2.2661
	HP0131	Predicted gene	1.7324
	HP0132	sdaA	1.2527
	HP0140	lctP	1.1428
	HP0142	mutY	1.0653
	HP0143	Predicted gene	1.0272
	HP0230	kdsB	1.2419
	HP0260	mod	1.4971
	HP0261	Predicted gene	1.1733
	HP0262	Predicted gene	1.128
	HP0263	hpaim	1.3069
	HP0328	lpxK	1.5594
	HP0329	nadE	1.03
	HP0342	Predicted gene	1.4897
	HP0343	Predicted gene	2.741
	HP0346	Predicted gene	1.4341
	HP0366	Spore coat polysaccharide biosynthesis protein C	2.1667
	HP0367	Predicted gene	1.6527
	HP0373	Predicted gene	2.8507
	HP0388	tRNA methyltransferase	1.2813
	HP0394	Predicted gene	1.0201
	HP0428	Predicted gene	2.0485
	HP0430	Predicted gene	1.7632
	HP0431	ptc1	2.2646
	HP0432	Predicted gene	2.4158
	HP0434	Predicted gene	2.3603
	HP0440	topA	1.307
	HP0441	VirB4 homolog	1.5219
	HP0453	Predicted gene	1.505
	HP0462	hsdS	1.5466
	HP0463	hsdM	1.5851
	HP0472	omp11	1.916
	HP0483	Predicted gene	1.0174
	HP0522	cag3	1.0262
	HP0524	cag5	1.5355
	HP0525	cagα	1.6885
	HP0526	CagZ	1.2767
	HP0527	cag7	2.8708
	HP0538	cag17	1.4644
	HP0543	cag22	1.1474
	HP0601	flaA	3.1409
	HP0602	Endonuclease III	1.881
	HP0603	Predicted gene	2.5156
	HP0611	Predicted gene	1.6473
	HP0613	ABC transporter ATP-binding protein	1.1885
	HP0621	mutS2	1.1332
	HP0638	Membrane protein	1.1037
	HP0651	Fucosyltransferase	1.4555
	HP0652	serB	1.9143
	HP0666	glpC	1.321
	HP0673	Predicted gene	1.131
	HP0675	xerC	1.6417
	HP0690	fadA	1.1721
	HP0711	Predicted gene	1.6394
	HP0713	Predicted gene	2.6885
	HP0728	Predicted gene	1.2074
	HP0751	flaG	2.6091
	HP0752	fliD	2.6449
	HP0753	fliS	1.2148
	HP0754	5-formyltetrahydrofolate cyclo-ligase	2.0228
	HP0755	Predicted gene	1.3991
	HP0757	Beta-alanine synthetase homolog	1.2021
	HP0758	Membrane protein	1.8156
	HP0759	Membrane protein	1.6024
	HP0821	uvrC	1.3838
	HP0846	hsdR	1.8459
	HP0860	gmhB	1.369
	HP0896	omp19	1.8089
	HP0897	Predicted gene	1.124
	HP0922	Membrane protein	1.2871
	HP0939	yckJ	1.4377
	HP0941	alr	1.331
	HP0942	dagA	1.1314
	HP0943	dadA	1.293
	HP0985	Predicted gene	1.0004
	HP1000	Para	1.4507
	HP1002	Predicted gene	1.3929
	HP1017	rocE	1.704
	HP1020	ispDF	1.2427
	HP1021	cheY	1.6902
	HP1022	Predicted gene	1.0345
	HP1027	fur	1.1997
	HP1047	rbfA	1.0606
	HP1051	Predicted gene	1.1447
	HP1080	Membrane protein	1.3598
	HP1081	Predicted gene	1.5337
	HP1095	tnpB	1.4743
	HP1119	flgK	3.4284
	HP1120	Predicted gene	3.1475
	HP1121	BSP6IM	1.8938
	HP1148	trmD	1.1276
	HP1165	tetA	1.2466
	HP1167	Predicted gene	4.0239
	HP1215	Predicted gene	1.2189
	HP1233	Predicted gene	2.281
	HP1238	amiF	1.387
	HP1258	Predicted gene	1.0603
	HP1321	ATP-binding protein	1.45
	HP1390	Predicted gene	3.3507
	HP1391	Predicted gene	1.915
	HP1440	Predicted gene	3.7316
	HP1505	Predicted gene	1.123
	HP1519	Predicted gene	2.1358
	HP1523	recG	1.2919
	HP1589	Predicted gene	1.091
	HPr05	HPrrnB5S	1.2855
	HPt01	tRNA-Glu-1	1.7745
	HPt08	tRNA-Asn-1	1.3011
	HPt25	tRNA-Ser-1	1.3979
	HPt26	tRNA-Pro-1	1.697
	HPt36	tRNA-Phe-1	2.3394
Downregulated	HP0003	kdsA	−1.0474
	HP0004	icfA	−1.4707
	HP0010	groEL	−1.7937
	HP0011	groES	−1.2866
	HP0015	Predicted gene	−1.0676
	HP0033	clpA	−1.0831
	HP0035	Predicted gene	−1.1771
	HP0036	Predicted gene	−1.1712
	HP0057	Predicted gene	−2.2975
	HP0072	ureB	−1.48
	HP0073	ureA	−1.5839
	HP0091	hsdR	−1.0385
	HP0099	tlpA	−1.1019
	HP0100	Predicted gene	−1.0583
	HP0102	Predicted gene	−1.5088
	HP0103	tlpB	−1.5548
	HP0109	dnaK	−1.8288
	HP0110	GrpE	−1.142
	HP0111	Predicted gene	−1.1914
	HP0118	Predicted gene	−1.3158
	HP0145	fixO	−1.0377
	HP0153	recA	−1.5738
	HP0154	eno	−1.0973
	HP0157	aroK	−1.7674
	HP0213	gidA	−2.3523
	HP0229	omp6	−1.3943
	HP0243	napA	−1.9605
	HP0289	ImaA	−1.3117
	HP0290	lysA	−1.5474
	HP0291	Predicted gene	−1.2657
	HP0292	Predicted gene	−1.0587
	HP0294	amiE	−2.2148
	HP0296	rplU	−1.4814
	HP0297	rpmA	−1.4635
	HP0298	dppA	−4.9784
	HP0299	dppB	−1.4717
	HP0300	dppC	−2.088
	HP0301	dppD	−2.4619
	HP0302	dppF	−2.0374
	HP0303	obgE	A-3.1174
	HP0304	Predicted gene	−2.0838
	HP0318	Predicted gene	−1.2903
	HP0364	nrdF	−1.1763
	HP0377	dsbC	−1.3781
	HP0378	ycf5	−1.1151
	HP0390	tagD	−2.3061
	HP0415	Predicted gene	−2.9304
	HP0514	rplI	−1.1937
	HP0515	hslV	−1.2219
	HP0609	Predicted gene	−1.0705
	HP0616	cheV	−1.1044
	HP0620	ppa	−1.0328
	HP0625	ispG	−1.0162
	HP0630	mdaB	−1.6352
	HP0641	Predicted gene	−1.6672
	HP0663	aroC	−1.1477
	HP0677	Predicted gene	−1.0215
	HP0680	nrdA	−1.1181
	HP0681	Predicted gene	−1.0098
	HP0682	Predicted gene	−2.3295
	HP0686	fecA	−1.1601
	HP0696	*N*-methylhydantoinase	−1.1476
	HP0718	Predicted gene	−1.2217
	HP0761	Predicted gene	−1.1174
	HP0777	pyrH	−1.6091
	HP0783	Predicted gene	−1.133
	HP0789	Predicted gene	−1.0537
	HP0807	fecA	−1.1454
	HP0810	rsmD	−1.102
	HP0811	Predicted gene	−2.0849
	HP0824	ahpc	−1.201
	HP0829	guaB	−1.6093
	HP0830	gatA	−2.1157
	HP0834	engA	−1.0829
	HP0876	frpB	−2.1815
	HP0959	Predicted gene	−1.1905
	HP0960	glyQ	−1.6042
	HP0961	gpsA	−3.3854
	HP0983	Predicted gene	−1.2053
	HP1023	Predicted gene	−1.1999
	HP1029	Predicted gene	−1.2139
	HP1036	folK	−1.5392
	HP1037	Predicted gene	−1.2796
	HP1038	aroQ	−1.0187
	HP1055	Predicted gene	−1.3617
	HP1077	nixA	−1.3528
	HP1098	Predicted gene	−1.0557
	HP1114	uvrB	−1.6187
	HP1162	Predicted gene	−2.3026
	HP1164	trxB	−1.1085
	HP1177	hopQ	−1.657
	HP1180	nupC	−1.273
	HP1181	Multidrug transporter	−1.2283
	HP1186	arsR	−1.2884
	HP1212	atpE	−1.662
	HP1283	Predicted gene	−1.4378
	HP1286	Predicted gene	−1.6787
	HP1288	Predicted gene	−2.66
	HP1289	Predicted gene	−1.3261
	HP1323	rnhB	−2.4111
	HP1324	Predicted gene	−1.2158
	HP1325	fumC	−1.2411
	HP1326	Predicted gene	−1.9589
	HP1327	Predicted gene	−2.4519
	HP1333	Predicted gene	−1.1022
	HP1334	Predicted gene	−1.3602
	HP1338	nikR	−1.3032
	HP1372	MreC	−1.0638
	HP1395	omp30	−1.8492
	HP1404	hsdS	−1.0096
	HP1420	fliI	−1.2893
	HP1459	Predicted gene	−1.0261
	HP1465	HI1087	−1.3993
	HP1468	ilvE	−2.3918
	HP1469	omp31	−1.6033
	HP1483	ubiE	−1.1526
	HP1484	Membrane protein	−1.0726
	HP1487	Predicted gene	−1.0692
	HP1488	Predicted gene	−1.0942
	HP1496	ctc	−1.1262
	HP1501	omp32	−1.6835
	HP1508	Ferredoxin-like protein	−1.4878
	HP1512	Predicted gene	−1.685
	HP1526	lexA	−1.0048
	HP1534	TnpB	−1.2819
	HP1550	secD	−1.4823
	HP1551	yajC	−1.1451
	HP1561	ceuE	−1.1216
	HP1563	tsaA	−1.7381
	HP1582	pdxJ	−2.4696
	HP1583	pdxA	−1.7959
	HP1588	Predicted gene	−3.0945
	HPt06	tRNA-Val-2	−1.1089
	HPt07	tRNA-Ser-3	−2.4847
	HPt19	tRNA-Arg-4	−1.6638
	HPt27	tRNA-Ser-2	−1.0814
	HPt34	tRNA-Leu-2	−1.6756

### The Dpp Transporter Activates the Expression of CagA

**FIGURE 3:**
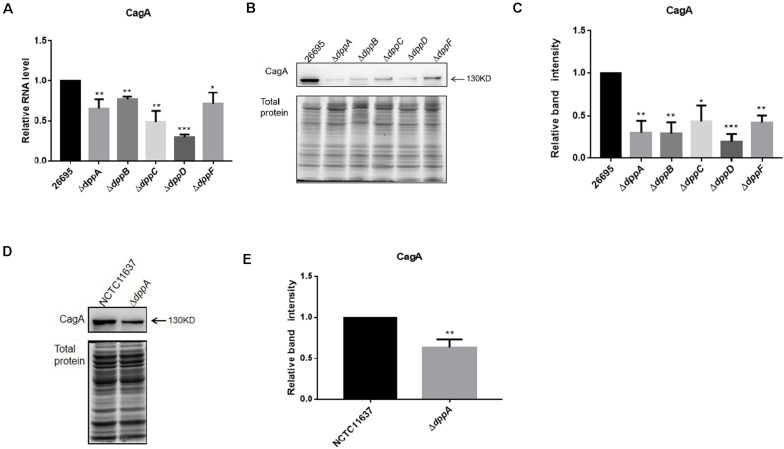
Effects of DppA on the expression of CagA. **(A)** mRNA level of CagA expressed in *H*. *pylori* 26,695 and Dpp transporter mutant strains. Values represent the relative mRNA level of CagA normalized to *H*. *pylori* 26,695. **(B–D)** CagA expression level determined by Western blot. Total protein represents the cell lysate resolved by SDS-PAGE. Protein bands representing CagA are indicated, and the position of a 130 kDa size marker is indicated by an arrow. **(C,E)** Quantification analysis of CagA bands. Densitometry was normalized to total protein. Values are shown as averages ± SD (*n* = 3). ***P < 0.001, **P < 0.01, *P < 0.05.

The transcriptomic study revealed differential expression of *H. pylori* virulence genes, especially those involved with *cag*PAI, between the wild-type and Δ*dppA* strain. In this study, we focused on those virulence factors that are closely related to the cellular inflammatory response. We first investigated the expression of CagA at both the mRNA and protein levels. The qPCR results showed that CagA mRNA levels in Δ*dppA*, Δ*dppB*, Δ*dppC*, Δ*dppD*, and Δ*dppF* strains were lower than in the *H. pylori* 26,695 wild-type strain (Figure 3A). CagA protein expression was also investigated, and similar results were obtained, i.e., CagA expression was repressed in Δ*dppA*, Δ*dppB*, Δ*dppC*, Δ*dppD*, and Δ*dppF* strains (Figures 3B,C). This was also shown in the NCTC11637 background, as CagA expression was significantly lower in Δ*dppA* compared with the wild type (Figures 3D,E). This result demonstrated that the Dpp transporter is important for the expression of CagA, and that DppA, DppB, DppC, DppD, and DppF are all critical for the function of the Dpp transporter to regulate the expression of CagA.

### The Dpp Transporter Causes the Inhibition of the Expression of Genes Encoding T4SS

**FIGURE 4:**
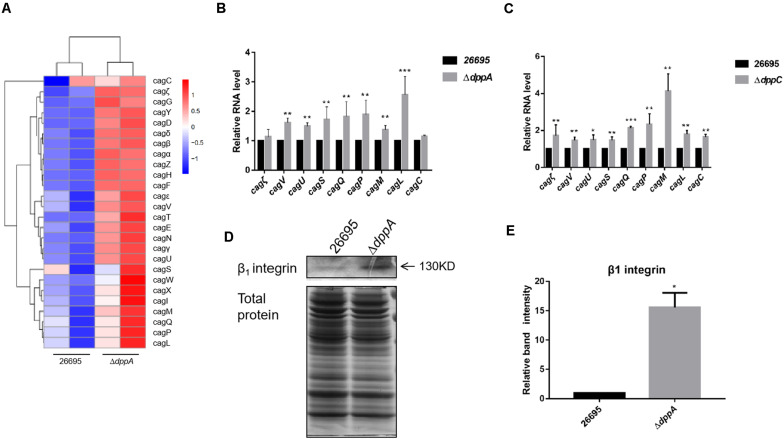
Effects of DppA on the expression of CagT4SS. **(A)** Hierarchical cluster analysis of T4SS gene expression in 26,695 and Δ*dppA* strains. **(B)** Determination of mRNA levels of T4SS components in *H*. *pylori* 26,695, Δ*dppA*, and Δ*dppC* **(C)**. Values represent the relative mRNA level of each gene normalized to *H*. *pylori* 26,695. **(D)** α5β1 integrin bound by *H. pylori* 26,695 and Δ*dppA*. Bands representing β1 integrin are indicated, and total bacterial protein load is shown. **(E)** Quantification analysis of β1 integrin bands. Densitometry was normalized to total protein. ***P < 0.001, **P < 0.01, *P < 0.05.

Our RNA sequencing data suggested that components of T4SS, including Cag3, Cag5, Cagα, CagZ, Cag7, and Cag22, were upregulated in Δ*dppA* mutants (Table 2). Thus, we analyzed all 26 genes related to T4SS; surprisingly, most of these genes were expressed relatively highly in Δ*dppA* mutants (Figure 4A). Next, we performed qPCR to confirm this result. T4SS genes comprise nine operons, according to a previous study (Kabamba et al., 2018). To evaluate the effects of Dpp on the expression of T4SS genes, we compared the mRNA levels of Cagζ, CagV, CagU, CagS, CagQ, CagP, CagL, CagY, CagM, CagE, and CagC from each operon. Except Cagζ and CagC, whose expression showed no difference between wild type and Δ*dppA*, the expression of CagV, CagU, CagS, CagQ, CagP, CagM, and CagL was significantly higher in Δ*dppA* compared with *H. pylori* 26695 (Figure 4B). This was consistent with our transcriptomic data. We also tested the Δ*dppC* background, and found that all 10 genes from each operon were expressed more in Δ*dppC* than in wild-type strain. During infection, CagA or LPS metabolites were delivered through the T4SS to gastric epithelial cells, and this was dependent on the direct interaction between T4SS proteins and α5β1 integrins (Kwok et al., 2007). To investigate if the Dpp transporter also influenced the binding of T4SS to α5β1 integrin, a bacterial pulldown assay using purified α5β1 integrin was performed, and we measured the amount of α5β1 integrin bound by *H*. *pylori*. The results showed that the Δ*dppA* strain bound significantly more α5β1 integrin compared with *H. pylori* 26,695 wild-type strain (Figures 4D,E). These results suggested that deficiency in Dpp transporter resulted in a higher expression of T4SS genes and an increase in T4SS binding to α5β1 integrin.

### The Dpp Transporter Causes Lower Expression of Outer Membrane Proteins and Reduces the Adhesion of *H. pylori* to AGS Cells

**FIGURE 5:**
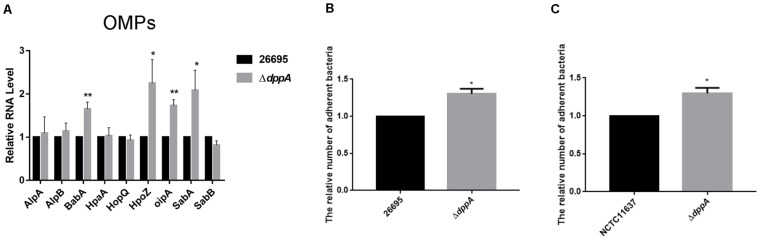
Effects of DppA on the adherence of *H. pylori* to AGS cells. **(A)** qPCR study of the expression of OMPs in *H. pylori* 26,695 and its isogenic mutant Δ*dppA.* Adherence of *H*. *pylori* to AGS cells. Relative adherence represents the number of Δ*dppA* cells adherent to AGS cells normalized to *H. pylori* 26,695 **(B)** and NCTC11637 **(C)**. Data shown are the average values from three independent experiments, and bars represent standard deviations. **P < 0.01, *P < 0.05.

Our transcriptomic study revealed that several outer membrane proteins related to adhesion were differentially expressed in Δ*dppA* strain. This suggested that the Dpp transporter might play an important role in bacterial adhesion. To test this hypothesis, we first confirmed the expression of the OMPs involved in bacterial adhesion. Our results showed that, compared with *H. pylori* 26,695, the expression of adhesion genes (*babA*, *hopZ*, *oipA*, and *sabA*) was higher in a Δ*dppA* strain, while *alpAB*, *hpaA*, *hopQ*, and *sabB* showed similar expression levels between the wild-type and Δ*dppA* strains (Figure 5A). This suggested that DppA caused a lower expression of OMPs. We also investigated the expression of OMPs in Δ*dppB*, Δ*dppC*, Δ*dppD*, and Δ*dppF* strains, and showed higher expression of BabA, HopZ, OipA, and SabA than in wild-type (data not shown), which confirmed that the Dpp transporter causes a lower expression of OMPs. Next, to verify whether the Dpp transporter altered the adhesion of *H. pylori* to AGS cells, AGS cells were infected with *H*. *pylori* 26,695 wild-type and Δ*dppA* cells; subsequently, we investigated the number of bacteria bound to AGS cells. Our results showed that Δ*dppA* cells had a higher binding capacity compared with wild-type 26,695 cells (Figure 5B). *H*. *pylori* NCTC11637 and its isogenic mutant Δ*dppA* were also analyzed, and we found that in the *H*. *pylori* NCTC11637 strain, deletion of *dppA* also resulted in a higher bacterial adhesion level (Figure 5C). This suggested that in *H. pylori*, the Dpp transporter caused a reduced expression level of OMPs, including *babA*, *hopZ*, *oipA*, and *sabA*, thereby reducing the adhesion of *H. pylori* to AGS cells.

### The Dpp Transporter Inhibits *H. pylori* Activation of Gastric Epithelial NF-κB

**FIGURE 6:**
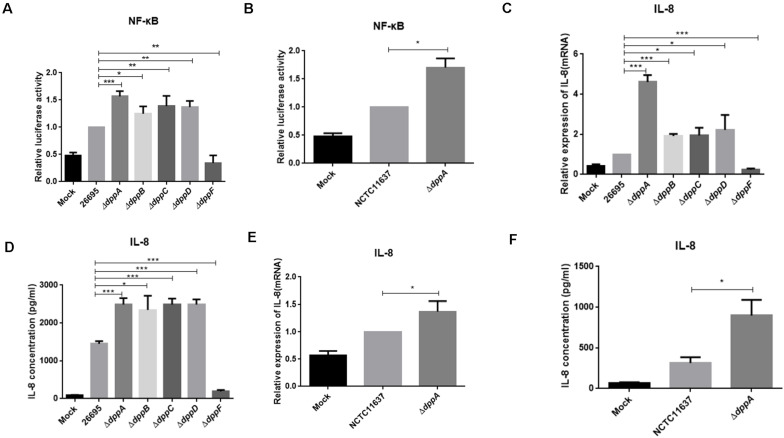
NF-κB activation and IL-8 production induced by *H*. *pylori* and Dpp transporter mutant strains. **(A)** NF-κB activity determined by luciferase activity of NF-κB-*luc*. AGS cells were transfected with a NF-κB luciferase reporter and were infected with *H. pylori* 26695, Δ*dppA*, Δ*dppB*, Δ*dppC*, Δ*dppD*, or Δ*dppF*, as well as being infected with *H*. *pylori* NCTC11637 and its isogenic mutant Δ*dppA* **(B)**. Relative luciferase activity represents luciferase activity normalized to mock samples. **(C,E)** Expression of IL-8 in AGS cells as determined by qPCR or by ELISA **(D,F)**. AGS cells were infected with *H*. *pylori* 26,695 and its isogenic Dpp transporter mutant strains, or *H. pylori* NCTC11637 and Δ*dppA* for 4 h with an MOI of 100. Data represent averages normalized to mock controls and are shown as the mean ± SD (*n* = 3). ***P < 0.001, **P < 0.01, *P < 0.05.

Upon adhesion to AGS cells, *H*. *pylori* directly activates NF-κB through the T4SS, which delivers the effector protein CagA, peptidoglycan, or ADP-heptose to cells. We investigated the effect of the Dpp transporter on *H. pylori*-induced NF-κB activation in AGS cells. We performed a dual-luciferase reporter assay using an NF-κB-*luc* reporter plasmid. After 4 h of infection with *H. pylori*, NF-κB was activated in wild-type infected cells. We also found that Δ*dppA*, Δ*dppB*, Δ*dppC*, and Δ*dppD* infection activated NF-κB to a level 50% higher than infection with a wild-type strain (Figure 6A). However, infection with the Δ*dppF* strain failed to activate NF-κB, likely due to low activity of Δ*dppF* for its low growth ability as shown in Figure 1E. We also checked *H*. *pylori* NCTC11637 and its isogenic mutant Δ*dppA*, and found that NF-κB-*luc* was expressed at a level 70% higher than in wild type (Figure 6B). This suggested that the Dpp transporter in *H*. *pylori* reduced the ability to activate NF-κB in AGS cells. Activation of NF-κB directly induces the expression of the inflammatory factors, including IL-8 (Brandt et al., 2005). Thus, we next utilized by ELISA and qPCR to examine IL-8 expression in AGS cells infected by *H*. *pylori*. Our results showed that IL-8 expression in AGS cells induced by *H. pylori* Δ*dppA*, Δ*dppB*, Δ*dppC*, and Δ*dppD* was significantly higher than that induced by wild-type *H. pylori* 26,695 (Figures 6C,D). As shown in Figure 6A, Δ*dppF* failed to activate the expression of IL-8 in AGS cells. In *H. pylori* 11,637, Δ*dppA* also induced a higher level of IL-8 expression compared with wild-type *H. pylori* NCTC11637 (Figures 6E,F). This indicated that the Dpp transporter repressed NF-κB and IL-8, thereby reducing the inflammatory response of AGS cells induced by *H*. *pylori*.

Taken together, this work was the first study to show the role of the Dpp transporter in the regulation of virulence of *H*. *pylori*. Our study demonstrated that the Dpp transporter is important for the growth of *H*. *pylori*, suggesting that dipeptides might serve as an important nutrient source for this bacterium. Although Dpp transporter-deficient strains proliferated slower, they were associated with higher bacterial adhesion and T4SS expression and induced a stronger inflammatory response in AGS cells. The Dpp transporter also activated the expression of CagA, illustrating the complex role of Dpp transporters in subtle control of bacterial virulence.

## Discussion

The host tissue is a rich source of nutrients for bacteria, providing nutrients such as sugars and amino acids. To acquire the nutrients from host, pathogens produce specific virulence factors and causes host damage. It is important to understand the interaction between metabolism and bacterium pathogenesis since bacterial growth is the main goal for the pathogen to colonize in the host (Rohmer et al., 2011). Peptide transporters are important to acquire carbon from host sources for pathogen’s growth. Moreover, these transporters are also responsible for importing environmental cues to coordinate bacterial behavior (Garai et al., 2017). Human pathogens always face various environmental stresses, such as temperature variation, pH, nutrient changes, and oxidative stress (Shao et al., 2005). Understanding these transporters and their cognate substrates may help in unraveling the mechanisms of bacterial adaptation through changes in bacterial behavior, including virulence.

In this study, we found that the Dpp transporter was important for the bacterial growth (Figure 1). However, *H*. *pylori* Δ*dppD* and Δ*dppF* grew significantly slower compared to wild type, Δ*dppA*, Δ*dppB*, or Δ*dppC* cells. DppD and DppF are both dipeptide ABC transporter ATP binding subunits, which suggests that Δ*dppD* and Δ*dppF* might completely abolish the function of Dpp transporters, resulting in a shortage of nutrients. Specifically, we noticed that the Δ*dppF* strain grew much slower compared with other strains. DppF acts as an ABC transporter ATP binding subunit, which might be critical for the growth of *H*. *pylori*, causing Δ*dppF* with its decreased capacity to induce an inflammatory response in AGS cells (Figures 6A,C,D).

In this study, we found that the CagA expression was largely dependent on the expression of the Dpp transporter (Figure 3). During the infection of gastric epithelial cells, *H. pylori* translocates CagA using the T4SS. Through interactions with SH2 domains, CagA activates their function to promote the Ras-Erk signaling pathway to activate oncogenesis of gastric epithelial cells (Tohidpour, 2016; Hatakeyama, 2017; Naumann et al., 2017). The CagA protein is strongly associated with development of gastric cancer, and regulation of CagA expression is closely related to gastric cancer development (Hatakeyama, 2017). Studies have shown that CagA expression varies depending on the growth stage and conditions (Karita et al., 1996). It has also been shown that high-salt concentrations induce the expression of CagA, which is related to gastric cancer development. Iron and pH also regulate CagA expression (Odenbreit et al., 1999). Our study provided new evidence on the regulation of CagA expression in *H*. *pylori*, suggesting that the nutrient status of the environment affects CagA expression and *H*. *pylori*-related gastric cancer. Specifically, when grown in environments with abundant nutrients, *H*. *pylori* might express high levels of CagA.

In the early stages of infection, *H. pylori* activates NF-κB in a CagT4SS-dependent manner. The regulation of T4SS expression might contribute to *H*. *pylori*-induced inflammatory response in AGS cells. In this study, we found upregulated expression of T4SS components in the Δ*dppA* strain (Figures 4A,B), which was also replicated in a Δ*dppC* strain (Figure 4C). Among these genes, RNA sequencing data and qPCR results showed that genes were upregulated in Δ*dppA* strain to a different degree, which suggested that although these genes are all responsible for the T4SS apparatus, they might be regulated by different mechanisms. A previous study investigated the expression of T4SS and found that these genes responded differently to growth phase, temperature, pH, iron, and cell contact (Yamaoka et al., 2000). Some environmental signals even exert pleiotropic effects on these genes. This suggests that T4SS expression and assembly are controlled by sophisticated mechanisms, but more studies are necessary. The T4SS machinery translocates CagA, ADP-heptose, peptidoglycan, and other substrates to host cells (Mobley et al., 1991; Peck et al., 1999; Pfannkuch et al., 2019). Recent studies have shown that ADP-heptose is a novel pathogen-associated molecular marker in *H*. *pylori*, and it is the main factor activating NF-κB in a T4SS-dependent manner (Pfannkuch et al., 2019). We speculate that T4SS expression is repressed under rich nutrient conditions, and *H*. *pylori* reduces the translocation of ADP-heptose or other effector molecules. Under poor nutrient condition, T4SS expression is activated and results in a high level of activation of the NF-κB response.

After *H. pylori* passes through the mucous layer and reaches the gastric mucosa via flagellar movements, OMPs promote close contact between *H. pylori* and gastric epithelial cells. OMPs play important roles in the establishment of colonization (Yuichi et al., 2017; Terbenc et al., 2019). The OMPs in *H*. *pylori* have been gradually unveiled, and their cognate interaction partners have been identified. In this study, we found that expression of BabA, HopZ, OipA, and SabA, and bacterial adhesion were upregulated in the Δ*dppA* strain (Figure 6). BabA was the first OMP identified to be involved in the adhesion of *H*. *pylori* and important for inducing severe inflammation in the stomach. Moreover, studies have shown that T4SS function and CagA translocation are enhanced by BabA (Boren et al., 1993; Ilver et al., 1998; Aspholm-Hurtig et al., 2004). Some studies have shown that the adhesion ability is significantly decreased in *oipA* mutant strain and *hopZ* mutant strain in AGS cells (Peck et al., 1999; Dossumbekova et al., 2006). SabA is also important for colonization and induction of inflammation in the stomach (Aspholm et al., 2006). SabA expression is regulated by a pH-responsive ArsRS two-component signal transduction system (Goodwin et al., 2008). The HopZ gene is involved in the adhesion of *H*. *pylori* to gastric epithelial AGS cell line *in vitro*, but it did not show any influence on the ability of colonization in the stomachs of guinea pigs (Peck et al., 1999). However, the cognate receptor of HopZ remains unknown. Our study suggested that the Dpp transporter in *H. pylori* plays an important role in the colonization of the stomach.

Besides the virulence factors investigated in this study, our transcriptomic study by RNA sequencing also indicated that the expression of other virulence genes is also altered in the Δ*dppA* background. Flagellar coding genes, including *flaA*, *flaB*, *fliD*, *flaG*, and *flgK*, were significantly upregulated in Δ*dppA* cells (Table 2). Flagellar movement is critical for the initial colonization of *H. pylori* by penetrating gastric mucus layer (Gu, 2017). Indeed, flagellar movement of *H. pylori* is an important factor in mediating high density colonization and severe inflammation. Studies have shown that FlaA and FlaB are necessary for *H. pylori* colonization of animals (Josenhans et al., 1995). ADP-heptose is a lipopolysaccharide synthesis intermediate, which is responsible for *H. pylori*-induced NF-κB activation (Pfannkuch et al., 2019). RNAseq data showed that upregulated expression of majority of LPS-related metabolic genes and the ADP-heptose synthesis gene *gmhB* in the Δ*dppA* strain (Table 2). GmhB (Hp0860) is an important synthase gene for the synthesis of ADP-heptose by dephosphorylation of D-*glycero*-β-d-*manno*-heptose-1,7-bisphosphate (HBP) (Stein et al., 2017). This suggests that LPS synthesis and ADP-heptose production might be upregulated in Δ*dppA*, thereby enhancing *H*. *pylori*-induced IL-8 production and NF-κB activation.

In conclusion, we have demonstrated that the Dpp transporter affects the expression of virulence factors such as CagA, T4SS, and OMPs. The Dpp transporter might enable the bacteria to recognize environmental nutrient conditions and change virulence factors such as adhesion and stimulate the release of other virulence factors. Since *H*. *pylori* causes a chronic infection and is closely related to gastric cancer, our study suggests that when nutrients are limited, and the Dpp transporter fails to transport dipeptides, *H*. *pylori* enhances its ability to colonize and stimulates an inflammatory response to acquire nutrients from the host. Thus, *H*. *pylori* tends to repress its ability to stimulate an inflammatory response in gastric epithelial cells while delivering the oncoprotein CagA, which induces gastric cancer.

## Data Availability Statement

The original contributions generated for this study are publicly available. This data can be found here: NCBI Gene Expression Omnibus database (GEO; http://www.ncbi.nlm.nih.gov/geo) under accession number GSE16421 (www.ncbi.nlm.nih.gov/geo/query/acc.cgi?acc=GSE164216).

## Author Contributions

YW and FS designed the study. XX, JC, and SF performed the experiments. XH analyzed the data. YW, XX, and FS wrote the manuscript. All authors have read and approved the submitted version.

## Conflict of Interest

The authors declare that the research was conducted in the absence of any commercial or financial relationships that could be construed as a potential conflict of interest.

## Footnotes

1 http://www.ncbi.nlm.nih.gov/geo
